# Long noncoding RNA functionality in imprinted domain regulation

**DOI:** 10.1371/journal.pgen.1008930

**Published:** 2020-08-06

**Authors:** William A. MacDonald, Mellissa R. W. Mann

**Affiliations:** 1 Department of Pediatrics, University of Pittsburgh School of Medicine, Pittsburgh, Pennsylvania, United States of America; 2 Rangos Research Center, UPMC Children’s Hospital of Pittsburgh, Pittsburgh, Pennsylvania, United States of America; 3 Department of Obstetrics, Gynaecology and Reproductive Sciences, University of Pittsburgh School of Medicine, Pittsburgh, Pennsylvania, United States of America; 4 Magee-Womens Research Institute, Pittsburgh, Pennsylvania, United States of America; University of California Los Angeles, UNITED STATES

## Abstract

Genomic imprinting is a parent-of-origin dependent phenomenon that restricts transcription to predominantly one parental allele. Since the discovery of the first long noncoding RNA (lncRNA), which notably was an imprinted lncRNA, a body of knowledge has demonstrated pivotal roles for imprinted lncRNAs in regulating parental-specific expression of neighboring imprinted genes. In this Review, we will discuss the multiple functionalities attributed to lncRNAs and how they regulate imprinted gene expression. We also raise unresolved questions about imprinted lncRNA function, which may lead to new avenues of investigation. This Review is dedicated to the memory of Denise Barlow, a giant in the field of genomic imprinting and functional lncRNAs. With her passion for understanding the inner workings of science, her indominable spirit and her consummate curiosity, Denise blazed a path of scientific investigation that made many seminal contributions to genomic imprinting and the wider field of epigenetic regulation, in addition to inspiring future generations of scientists.

## Introduction

The *H19* RNA was the first long noncoding RNA (lncRNA) within the mammalian genome to be discovered, followed shortly by the X-inactive-specific transcript (*Xist*) lncRNA [1,2]. Following pioneering work by Denise Barlow’s as well as other laboratories, imprinted lncRNAs were found to play pivotal roles in regulating parental-specific expression of neighboring imprinted genes (see references marked in bold for Denise Barlow’s contributions to the field). Importantly, identification of imprinted lncRNAs genes and their role in epigenetic regulation has ignited the field of research into lncRNAs. Saliently, thousands of lncRNAs have been found in mammals, with robust research efforts aimed at understanding their functionality and mechanism of action [3–8], including in X-inactivation (see recent review on *Xist* lncRNA function [9]). In this Review, we will describe the state of knowledge about imprinted lncRNAs, their function, and, in the spirit of Denise Barlow, address as well as pose the many fascinating and outstanding questions remaining to be answered.

### Genomic imprinting

Genomic imprinting is an epigenetic mechanism whereby gene regulation depends on the sex of the transmitting parent [10]. Imprinted genes, which are governed by this mechanism, exhibit transcriptional silencing when inherited by one parent and active transcription when transmitted by the other parent. Often, imprinted genes reside together in clusters or imprinted domains (2 to 20 genes). Since expressed and repressed alleles of imprinted genes reside in the same nucleus, they must be governed by *cis*-regulatory mechanisms. One of the seminal discoveries in genomic imprinting was the identification of genetic elements called gametic differentially methylated regions (gDMRs) that harbor the “imprinting mark” acquired in oocytes and sperm [10]. These marks are subsequently inherited by embryos and offspring, directing parental-specific allelic expression. Deletions of various gDMRs, experimentally or naturally, have validated the gDMR as a master switch, which coordinately regulates the repressed or active state of multiple imprinted genes within an imprinted domain [11–16], thus, earning the name of imprinting center, imprinting control element, or imprinting control region (ICR). Saliently, within an imprinted domain, ICRs possess the “imprinting mark”, rather than genes per se [10]. As a result, Denise Barlow refined terminology in the field, classifying allelic expression as “maternally or paternally expressed or repressed” and not as “maternally or paternally imprinted.” Importantly, while less than 1% of genes in the mammalian genome are regulated by genomic imprinting [17,18], deletion or loss of imprinting at these genes can lead to lethality or cause a broad spectrum of effects, including growth, developmental, metabolic, and neurological phenotypes [See reviews 19, 20]. As such, genomic imprinting has become a central paradigm for understanding how epigenetic mechanisms control expression and repression of genes, gene clusters, and gene pathways involved in development, health, and disease.

### Imprinted lncRNAs

**Fig 1:**
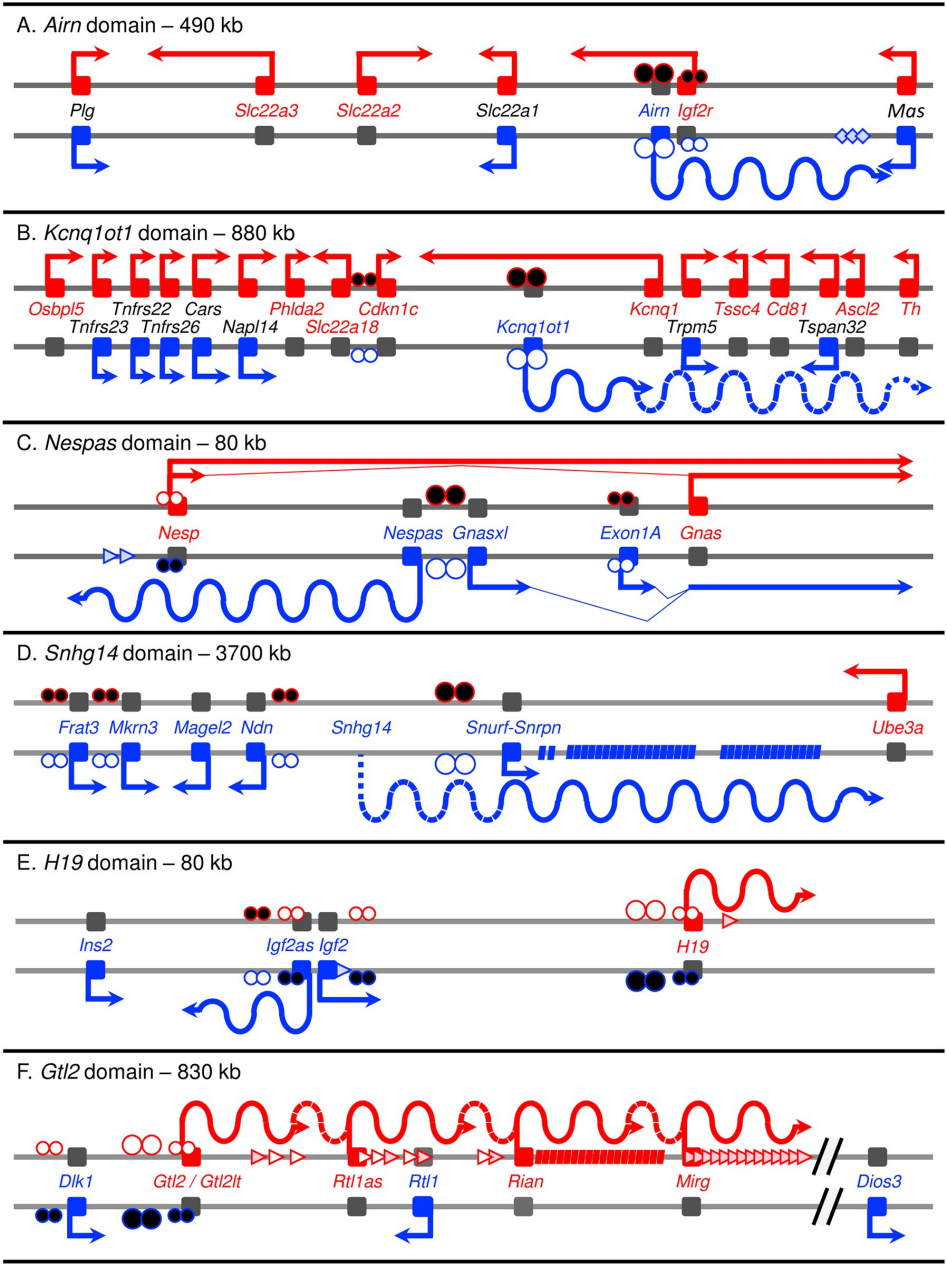
Imprinted domains and their long noncoding RNAs. (A) *Airn* imprinted domain. (B) *Kcnq1ot1* imprinted domain. (C) *Nespas* imprinted domain. (D) *Snhg14* imprinted domain. (E) *H19* imprinted domain. (F) *Gtl2* imprinted domain. Note: The domain sizes indicated likely represent minimum lengths of the domains [10]. Imprinted protein-coding genes, which are expressed from one parental allele while the other copy is silent, reside in clusters or imprinted domains. Maternally expressed genes are represented by a red arrow and text, while paternally expressed genes are represented by a blue arrow and text. Nonimprinted genes, where both alleles are expressed, also localize to imprinted domains (black text). At least one lncRNA (wavy line) is present with a domain (dashed wavy line represents potential extension of a lncRNA transcript). Regulation of imprinting across an imprinted domain is through a *cis*-acting mechanism that is controlled by an ICR (large circles) and its associated lncRNA gene. Note: Some imprinted genes in these domain exhibit tissue-specific imprinted expression. The DNA methylated state of ICRs is depicted by large black circles, while an unmethylated ICR is denoted by white circles. Smaller circles represent methylation at secondary, somatic DMRs. Small ncRNAs housed within lncRNAs are depicted as diamonds for endogenous small interfering RNAs, arrowheads for microRNAs, and rhomboids for snoRNAs.

Another seminal discovery in genomic imprinting was the identification of lncRNAs within most imprinted domains. As their name implies, lncRNAs are not known to code for proteins. In comparison to small noncoding RNAs, lncRNAs are distinguished by their long length (greater than 200 bp). Imprinted lncRNAs range in length from approximately 1.9 kb to approximately 1,000 kb, leading Barlow and colleagues to classify the very long imprinted lncRNAs as macro noncoding RNAs (ncRNAs) due to their extraordinary length [21]. Imprinted lncRNAs also differ from mRNAs in their capacity to be spliced. Long ncRNAs are spliced transcripts with a low intron to exon ratio that are present in the cytoplasm and/or are present predominantly as unspliced transcripts that are retained in the nucleus [4]. As a result, imprinted lncRNAs have short half-lives compared to mRNAs [22–28]. Another important aspect of imprinted lncRNAs is their intimate relationship to ICRs, with promoters either embedded within or closely positioned to the ICR. Generally, ICR-embedded promoters are maternally methylated, reflecting oocyte-specific acquisition of DNA methylation following transcription elongation and histone H3 lysine 36 trimethylation (H3K36me3) disposition [29–31], while lncRNA promoters near ICRs are paternally methylated, acquiring DNA methylation at intergenic sequences during spermatogenesis (Fig 1). Importantly, imprinted lncRNA expression is dependent on the ICR being in an unmethylated state. Another particular fascinating feature of nuclear lncRNAs is their accumulation as a large volume or “cloud” at the parental domain from which they are expressed. Here, imprinted lncRNAs generally function through *cis*-acting, long-range effects, silencing alleles of multiple neighboring genes, often bidirectionally, at megabase distances [32]. However, it is still uncertain whether transcription per se is a key feature of lncRNA function, with accumulation reflecting the lncRNAs unusual length and processing, or whether lncRNA accumulation is indicative of a physical role for lncRNAs in directing the silencing of neighboring imprinted genes [4,33]. Whichever the case, the mechanisms of repressive function are not yet completely understood. Below, we examine proposed mechanism for lncRNA function, focusing primarily on mouse models of the better-studied imprinted domains. For consistency, we have designated these domains according to the associated lncRNA gene: *Airn* (antisense of *Igfr2*; Fig 1A), *Kcnq1ot1* (*Kcnq1* opposite transcript 1; Fig 1B), *Nespas* (*Nesp* antisense; Fig 1C), *Snhg14* (*Small nucleolar RNA host gene 14*, also known as *Snrpnlt*, *Lncat*, *IC-Snurf-Snrpn*, *Snrpn-Ube3a*, *U-Ube3aast*, *Ube3aast* and *Ube3a-as*; Fig 1D), *H19* (Fig 1E), and *Gtl2* (also known as *Meg3*; Fig 1F).

## Functional output linked to lncRNA transcription

### Imprinted lncRNA gene transcription runs interference

How lncRNAs regulate parent-specific expression of multiple neighboring imprinted genes within imprinted domains is of major research interest. The simple act of transcription of a lncRNA is itself proposed to be a mechanism of action called transcriptional interference [34], where transcription of one gene directly suppresses transcription of a second gene in *cis* [35]. Transcriptional interference has been proposed for the *Airn* lncRNA gene in silencing *Igf2r* [36]. The *Airn* lncRNA gene is 108 to 118 kb in length with variable 3’ length [37], which originates from the *Airn* ICR within intron 2 of the *Igf2r* gene [38] (Fig 1A). This sets up a situation of convergent transcription of the paternal *Airn* lncRNA in an antisense direction across the paternal *Igf2r* promoter. Premature terminations of the *Airn* lncRNA before but not after the *Igf2r* promoter result in paternal *Igf2r* reactivation, suggesting that transcription through the promoter is the primary mechanism of paternal *Igf2r* silencing [36,39]. Furthermore, in embryonic stem cells (ESCs), inducible *Airn* transcription is both necessary and sufficient to silence paternal *Igf2r* expression [40]. Collectively, these findings established a clear role for transcriptional interference as a mechanism for silencing overlapping genes.

**Fig 2:**
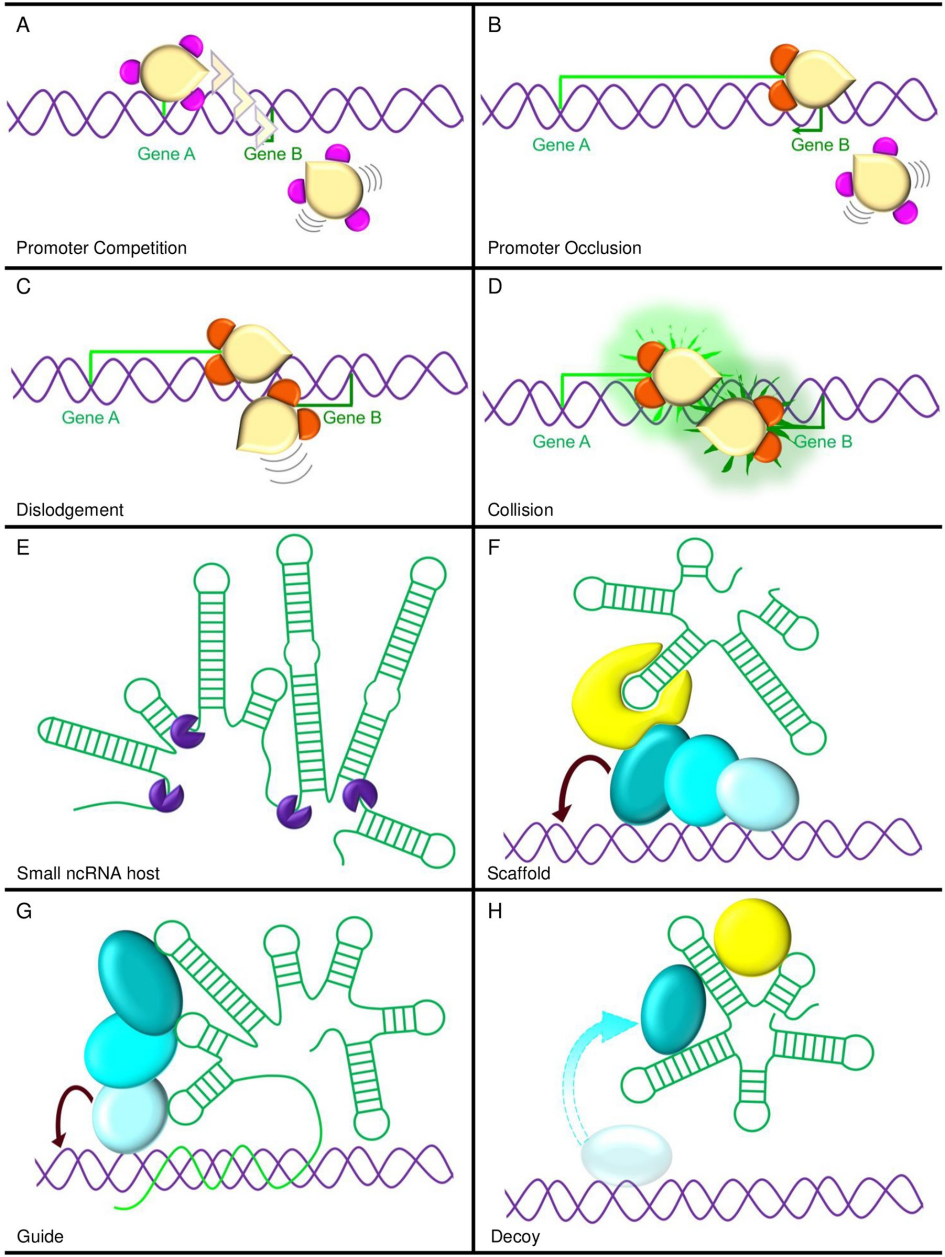
Function of long noncoding RNA genes. **(A-D)** Expression of lncRNAs run transcription interference by directly suppressing transcription of a second gene in *cis* through multiple modes of action. (**A**) Promoter competition involves a strong promoter suppressing recruitment of the transcription initiation complex at a weaker promoter. (**B**) Promoter occlusion arises when transcription of a lncRNA blocks recruitment of the transcription initiation machinery at an overlapping promoter. (**C**) Dislodgement results when a lncRNA engages the elongation complex faster, leading to dislocation of the transcription complex at another transcript. (**D**) Collision involves the crash of two convergent elongation complexes between the lncRNA transcript and a second transcript, leading to premature termination of the latter. (**E-H**) Long RNAs themselves also have direct functions in gene regulation. (**E**) Long ncRNAs serve as a host for small ncRNAs which are excised from the lncRNA transcript. (**F**) Scaffold function refers to lncRNAs acting as the framework for RNA-binding proteins and modifier complexes interactions with DNA and chromatin to carry out their function. (**G**) lncRNAs can function as guides by aligning with specific DNA sequences though lncRNA and DNA pairing, attracting chromatin modifiers and directing chromatin modification to target sequences. (**H**) Decoy function involves lncRNAs acting as a sink or sponge to sequester proteins away from a target site.

Several models have been proposed for this suppressive mechanism [35]. The first model involves promoter competition, where the stronger promoter, such as *Airn*, enables better recruitment of the transcription initiation complex than the weaker promoter, such as *Igf2r* (Fig 2A). Second is the promoter occlusion model, where one transcript, such as the *Airn* lncRNA, is more robustly and continuously expressed, causing the elongation complex to block the overlapping *Igf2r* promoter from recruiting the transcription initiation machinery (Fig 2B). The third model proposes dislodgement, where one transcript is faster at engaging the elongation complex, such as the *Airn* lncRNA, leading to dislocation of the transcription complex at the slower *Igf2r* promoter (Fig 2C). In addition to transcriptional interference between the paternal *Airn* and *Igf2r* alleles, a transcription interference mechanism has been proposed where transcription of the paternal *Airn* lncRNA would interfere with activators or enhancers within the *Airn* gene body, thereby preventing initiation and/or up-regulation of paternal, nonoverlapping upstream *Slc22a2* and *Slc22a3* alleles [32,41]. However, upon deletion of the entire *Airn* gene, no regulatory elements have been identified that lead to up-regulation of paternal *Slc22a2* and *Slc22a3* alleles [41]. This indicates that a transcription interference mechanism is not involved in long-range silencing of the *Airn* domain in *cis*, where transcription is nonoverlapping.

Another form of transcriptional interference is transcriptional collision, which by definition is limited to convergently transcribed genes. The collision model proposes the crash of two converging elongation complexes, leading to premature termination of one complex (Fig 2D) [35]. This mechanism has been proposed as a silencing mechanism within the *Snhg14* imprinted domain [42] (Fig 1D). The *Snhg14* lncRNA likely originates from exons upstream of the *Snurf*-*Snrpn* gene and extends approximately 1,000 kb through to the *Ube3a* gene [43,44]. In mouse neurons, the *Snhg14* lncRNA gene is paternally expressed, while the *Ube3a* gene is maternally expressed. Deletions of the *Snhg14* promoter or truncation of the paternal *Snhg14* lncRNA between *Snord115* and *Ube3a* result in reactivation of the paternally silenced *Ube3a* allele [42,45,46]. Since both parental *Ube3a* promoters engage the transcription initiation complex, transcriptional interference via promoter competition, promoter occlusion, and dislodgement are eliminated as mechanisms. Furthermore, the paternally expressed *Snhg14* lncRNA extends as far as the intronic region of *Ube3a* between exons 4 and 5, coinciding with the position of paternal *Ube3a* termination [42]. Thus, transcriptional collision of RNA polymerases on the paternal allele could account for paternal *Ube3a* transcription stalling, incomplete elongation, and subsequent degradation of the paternal *Ube3a* transcript [42]. In support of a collision model, treatment with topoisomerase inhibitors, which block unwinding of DNA during transcription, leads to reactivation of paternal *Ube3a*, where it is proposed that stalling of elongation complexes prevents *Snhg14* lncRNA transcription from extending through to *Ube3a* [47].

Another imprinted domain, *Kcnq1ot1*, may also function though a transcription interference mechanism (Fig 1B). Deletion or conditional deletion of the *Kcnq1ot1* ICR (also known as KvDMR1); deletion of the *Kcnq1ot1* promoter; and premature truncations of the paternal *Kcnq1ot1* lncRNA to less than 3 kb all result in loss of paternal *Kcnq1ot1* lncRNA expression and reactivation of paternally silent, protein-coding genes across the domain [48,49]. To differentiate between *Kcnq1ot1* ncRNA transcription interference and *Kcnq1ot1* lncRNA silencing function, posttranscriptional depletion of the *Kcnq1ot1* lncRNA via RNA interference was carried out in embryonic and extraembryonic stem cells. No effect was observed on paternal allelic silencing of protein-coding genes in the domain [50]. As RNA interference acts posttranscriptionally, this strengthens a function through transcriptional interference. However, data are conflicting regarding the length of the *Kcnq1ot1* lncRNA gene, extending from 83 [37], 91 [51], 121 [32,52], and 471 kb in length [50], suggesting that the *Kcnq1ot1* lncRNA is at least 83 kb with a variable 3’ end (Fig 1B) and that transcriptional length of the *Kcnq1ot1* lncRNA could reflect differences between cell types and developmental stages. As the *Kcnq1ot1* lncRNA originates from the ICR within intron 11 of the *Kcnq1* gene [53], a *Kcnq1ot1* lncRNA transcript of 90 to 121 kb would not overlap the convergent *Kcnq1* promoter, limiting transcriptional interference to a collision model that prematurely terminates the paternal *Kcnq1* transcript but not other downstream paternally silenced genes (Fig 2D). On the other hand, longer transcription of the *Kcnq1ot1* lncRNA gene up to 471 kb would extend through five paternally repressed alleles, *Kcnq1*, *Tssc4*, *Cd81*, *Ascl2*, and *Th* [50]. All transcriptional interference mechanisms by the *Kcnq1ot1* lncRNA may apply to the four convergently transcribed genes, while transcription dislodgement or occlusion would be possible mechanisms in paternal *Th* silencing (Fig 2A–2D). Finally, a posttranscriptional mechanism involving the RNA inference pathway has been ruled out for *Kcnq1ot1* domain regulation following *Dicer* deletion [51].

*Nespas* is another imprinted domain that may operate though transcriptional interference (Fig 1C). The *Nespas* lncRNA gene is approximately 30 kb in length [28] (Fig 1C). The *Nespas* promoter is embedded within the *Nespas* ICR (also known as *Nespas-Gnasxl* gDMR), and when unmethylated on the paternal allele, *Nespas* is expressed [12]. *Nesp* and *Nespas* are convergently transcribed and overlapping sense-antisense genes. Paternal truncation of *Nespas* to 12.6 kb, after the *Nesp* gene and the *Nesp* somatic DMR, elicits no change in allelic expression or methylation of the paternal *Nesp* allele [28]. However, two additional paternal truncations, prior to the *Nesp* promoter at less than 100 bp, and just short of *Nesp* exon 2 at 10 kb, produce a loss of secondary methylation at the paternal *Nesp* DMR and a gain in H3K4me3 [28,54,55]. This indicates that *Nespas* lncRNA transcription across the *Nesp* promoter and DMR is required for a gain of de novo paternal methylation at the *Nesp* somatic DMR, likely via H3K36me3 deposition [54]. However, only the very premature *Nespas* truncation at 100 bp reactivated the silent paternal *Nesp* allele, supporting a mechanism of *Nespas* lncRNA collision of *Nesp* transcription between the promoter and exon 2 of the *Nesp* gene (Fig 2D). Both *Nespas* truncations also reduced expression of the paternal *Gnasxl* allele, likely through loss of *Nesp* DMR methylation [28,54,55]. Finally, only the very premature truncation leads to paternal silencing of *Exon1A* and paternal reactivation of the *Gnas* promoters, connecting paternal *Nesp* silencing with paternal-specific regulation of these genes.

Going forward, comprehensive studies are required to determine the exact mechanisms of specific transcriptional interference models at imprinted domains. How is transcriptional interference specifically mediated at the DNA sequence level? R-loops regulating the elongation complex have been proposed as one mechanism [47]. Do other structures such a G-quadraplexes play a role? Adding a twist (pun intended), how do these models function in the context of chromatin structure, since current models are mostly based on chromatin as a linear array?

## Functional output of posttranscriptional lncRNAs

### Imprinted lncRNAs as hosts

One function imprinted lncRNAs serve is as precursor transcripts for small regulatory RNAs (Fig 2E). Intriguingly, imprinted domains present a higher frequency of small ncRNAs compared to rest of the genome [56]. For example, within an intron of the mouse- and rat-specific, paternally expressed *Sfmbt2* gene alone, there is a large cluster of approximately 65 to 72 microRNAs (interspersed with B1 retrotransposons and microsatellite repeats) [57,58]. Imprinted lncRNAs themselves also host multiple small ncRNAs, and in some cases, they hold all the small ncRNAs within an imprinted domain [56]. Within the *Airn* lncRNA resides the *retro-Rangap1* (*Au76*) pseudogene, which harbors hairpin-loop structures that are processed into endogenous small interfering RNAs (485 sequences [59]; Fig 1A). Within the *Snhg14* lncRNA lie two large C/D type small nucleolar RNA (snoRNA) clusters, Snord115 (approximately 71 copies) and Snord116 (approximately 136 copies) plus 3 additional snoRNAs (Fig 1D) [60]. Situated within the *Nespas* and *H19* lncRNAs are 2 and 1 microRNAs, respectively [61,62] (Fig 1C and 1E). The approximately 220 kb-continuous, polycistronic *Gtl2lt* lncRNA (*Meg3-Rian-Mirg*), which includes the lncRNA-encoding sequences *Rtl1as* (microRNAs), *Rian* (C/D snoRNAs), and *Mirg* (microRNAs), contains one of the largest collections of small ncRNA clusters (approximately 40–50 microRNAs) within the genome (Fig 1F), followed by the *Snhg14* lncRNA [63–66]. Strikingly, an imprinted gene on human chromosome 19 is composed entirely of 46 microRNAs interspersed with Alu sequences, which are encoded by a single (or few) paternally expressed transcript [67]. While these small RNAs may have roles in regulating expression of multiple transcript targets [68], their action is through *trans*-acting function [69]. For example, the maternally expressed, *Rtl1as* ncRNA gene within the *Glt2* domain, which harbors two maternally expressed microRNAs (miR-127 and miR-136), regulates the dosage of the paternally expressed *Rtl1* gene [63,69–71], while miR-329 within the maternally expressed miR-379/miR-544 cluster (plus 6 other predicted miRs) regulates the paternally expressed *Dlk1* gene [65,72]. To date, no role has been identified for *cis*-regulated function in silencing alleles of neighboring imprinted genes [56]. That being said, it has been proposed that the repetitive nature of imprinted small ncRNA clusters may play a role in gene silencing [56]. Future studies will need to address whether the repetitive and 3D structural nature of small ncRNAs embedded within imprinted lncRNAs contribute to allelic silencing of adjacent imprinted genes.

### Imprinted lncRNAs as scaffolds

Transcriptional interference can account for suppressive function by lncRNA transcription for overlapping downstream genes. However, how lncRNAs silence upstream genes is still an outstanding question. A common feature of many imprinted lncRNAs is that transcripts localize as foci or “clouds” [4,73]. Given the short half-lives of imprinted lncRNAs [25–27], this would require a mechanism that is spatially driven. Thus, an alternative mechanism proposed for lncRNA function is as a scaffold for recruitment of chromatin modifiers to target promoters for allelic silencing [32,74]. In this capacity, lncRNAs act as a backbone onto which proteins are loaded to carry out their function [75,76] (Fig 2F). Within the paternal *Airn* domain, the *Airn* lncRNA accumulates and forms a RNA cloud on the paternal allele, spreading from the site of transcription to cover the upstream region containing the paternal silent *Slc22a2* and *Slc22a3* genes [77]. In trophoblast stem cells (TSCs), the paternal *Airn* lncRNA cloud associates with Polycomb repressive complex 1 (PRC1) and repressive modifications [H3K27me3, H4K20me1, H2AK119 monoubiquitination (u1)] [27]. In ESCs, PRC2 interacts with the *Airn* lncRNA [78]. Thus, it has been suggested that the *Airn* lncRNA recruits PRC2 and PRC1 to paternal alleles of imprinted genes within the domain, forming a repressive compartment [27,41]. In TSCs and visceral yolk sac endoderm, broad segments of parental-specific H3K27me3 and H2AK119u1 (20 kb sliding windows) stretch across a region that harbors the paternal *Slc22a3* and *Slc22a2* alleles [22,41]. Intriguingly, H3K27me3 levels at these broad segments appear to be dependent on *Airn* lncRNA levels, with overexpression increasing and reduced expression decreasing H3K27me3 levels in *cis* [22]. This points to a direct correlation between *Airn* lncRNA levels, H3K27me3 enrichment, and, potentially, PRC2 occupancy. In the placenta, the paternal *Airn* lncRNA may also acts as a scaffold with histone H3K9 methyltransferase, EHMT2 (also known as G9a) at the upstream paternal *Slc22a3* promoter region, thereby contributing to paternal *Slc22a3* silencing [77]. Premature truncation of the *Airn* lncRNA results in a loss of *Airn* lncRNA association with the *Slc22a3* promoter, EHMT2 is not recruited, and the paternal *Slc22a3* allele is reactivated [77]. Interestingly, the *Airn* lncRNA is not observed to recruit EHMT2 to the *Igf2r* promoter, reinforcing distinct mechanisms of action for the *Airn* lncRNA in paternal allelic silencing of imprinted genes within the domain [36,77]. Finally, HNRNPK (heterogeneous nuclear ribonucleoprotein K), an RNA-binding protein, may facilitate *Airn* lncRNA scaffold function. HNRNPK interacts with the *Airn* lncRNA, with its deletion reducing H3K27me3 across the paternal *Slc22a3* and *Slc22a2* alleles in TSCs [22]. As a model, HNRNPK may bind the *Airn* lncRNA and then interact with PRC2 through protein to protein interactions. DNA and/or histone binding domains in PRC2 proteins would mediate H3K27me3 modification across the paternal *Slc22a3* and *Slc22a2* alleles.

The paternal *Kcnq1ot1* lncRNA is also accredited with long-range repressive function of nine imprinted genes within the domain via scaffold functions (Fig 1B) [15,48,49,79]. As part of a lncRNA repressive function, the *Kcnq1ot1* lncRNA accumulates on or “coats” the paternal domain [27,50,51,80,81]. During early development, the paternal *Kcnq1ot1* imprinted domain harbors a contracted volume marked by PRC2 and PRC1 enrichment and repressive histone modifications (H2AK119u1, H3K9me3, and/or H4H20me1) [27]. In early embryos, stem cells and placentas, this is accompanied by repressive modifications (H3K27me3, H3K9me2/3, and/or H2AK119u1) at paternal alleles of upstream and downstream imprinted gene promoters [23,27,82–85] as well as broad segments of paternal-specific H3K27me3 and H2AK119u1 [22,41]. The *Kcnq1ot1* lncRNA also interacts with PRC2 and EHMT2 in placentas [52,86]. These findings have led to the claim that the paternal *Kcnq1ot1* lncRNA acts as a scaffold to recruit chromatin modifiers to target upstream and downstream gene promoters in *cis*, inducing their silencing by PRC2 and PRC1. An additional scaffold function for the *Kcnq1ot1* lncRNA has been postulated in postimplantation embryos, where the *Kcnq1ot1* lncRNA interacts with DNMT1 at the paternal *Cdkn1c* and *Slc22a18* loci, catalyzing DNA methylation at the paternal secondary *Cdkn1c*/*Slc22a18* DMRs [85]. Finally, the *Kcnq1ot1* lncRNA is bound by HNRNPK in TSCs and its deletion reduces H3K27me3 across the paternal *Kcnq1ot1* imprinted domain [22]. Thus, *Kcnq1ot1* lncRNA scaffold function may facilitate HNRNPK:PRC2:DNA/chromatin interactions, eliciting H3K27me3 bidirectionally across the paternal *Kcnq1ot1* imprinted domain.

At the *Nespas* imprinted domain, it is still unclear whether the *Nespas* lncRNA could play a role in domain regulation outside of transcription interference mechanisms (Fig 1C). However, two observation support *Nespas* scaffold function. Firstly, PRC2 has been found to interact with the *Nespas* lncRNA [78]. Secondly, premature termination of the *Nespas* lncRNA to less than 100 bp fails to repress the paternal *Nesp* allele, while truncation of the *Nespas* lncRNA at 10 kb retains paternal *Nesp* silencing [28,54,55]. These findings suggest that any scaffold function enabling repressive function of the *Nespas* lncRNA is contained within the first 10 kb of the *Nespas* lncRNA. Indeed, a major binding site for the PRC2 complex lies within 10 kb of the *Nespas* transcription start site [78]. Together, these data suggest a silencing role for the *Nespas* lncRNA through a scaffold function with PRC2.

Another imprinted domain for which a lncRNA may function as a scaffold is at the *Gtl2* imprinted domain (Fig 1F). The *Gtl2* lncRNA accumulates at the site of transcription, the maternal *Glt2* ICR (called IG-DMR) and the maternally silent *Dlk1* allele [24,87]. In ESCs, *Dlk1* is very lowly expressed from both the maternal and paternal alleles [24,87], with both showing EZH2 and H2K27me3 enrichment. The *Gtl2* lncRNA directly interacts with PRC2 and JARID2 [78,88]. Upon differentiation into embryoid bodies, cortical neurons or neuronal progenitor cells, *Dlk1* attains paternal-specific expression while the maternal allele remains repressed [24,87]. Deletions of the *Gtl2* promoter or gene body, as well as *Gtl2* depletion fail to repress the maternal *Dlk1* allele with reduced EZH2 and H3K27me3 enrichment [24,87]. Finally, *Ezh2* deletion also leads to lack of maternal *Dlk1* repression, albeit without a change in *Gtl2* lncRNA expression [87], suggesting that only scaffold functions of the Gtl2 lncRNA is perturbed by *Ezh2* deletion.

Many fascinating questions emerge from the above data. With respect to lncRNA coating of an imprinted domain, is the entire domain coated or only specific regions? If varying lncRNA levels lead to different levels of histone modifications, what are the mechanisms controlling lncRNA levels? Does lncRNAs scaffold function lead to lncRNA tethering to chromatin? Alternatively, is tethering an integral component of scaffold function and does it enable spreading of repression across the imprinted domain? Does the lncRNA form multiple scaffolds for numerous independent chromatin modifiers or does the scaffold exist as one large multimeric, macromolecular complex, executing multiple silencing mechanisms simultaneously?

### Imprinted lncRNAs as guides

As a guide, lncRNAs target specific DNA sequences though lncRNA and DNA pairing, attracting chromatin modifiers that directly modify target sequences (Fig 2G) [75,76,89]. As stated above, imprinted lncRNAs interact with neighboring imprinted genes in *cis*; the *Airn* lncRNA with the upstream paternal *Slc22a3* promoter; the *Kcnq1ot1* lncRNA with paternal promoters of at least six imprinted genes in the domain [52,85]; and the *Gtl2* lncRNA with the maternal *Dlk1* allele (Fig 1A, 1B and 1F) [24,87]. One mechanism for molecular guide function is through the lncRNA making contact with specific target genes in triplex lncRNA-DNA–DNA formation. In silico predictions in the human have revealed numerous triplex-forming DNA-binding sites within imprinted lncRNAs and target sites at promoters of imprinted genes, including the *AIRN* lncRNA to the IGF2R, SLC22A2, and SLC22A3 promoters; the *H19* lncRNA to multiple IGF2 promoters; and the *KCNQ1OT1* lncRNA to eight imprinted gene promoters in the domain [90]. In the mouse, multiple DNA-binding sites within the *Nespas* lncRNA have predicted triplex formation at matched target sites at the *Nesp*, *Gnasxl*, and *Gnas* promoters [90]. For the human *GTL2* imprinted domain, the secondary structure of in vitro- and ex vivo-produced *GTL2* lncRNA (approximately 1,600 nt) has been mapped for guide function [91]. The highly structured, *GTL2* lncRNA possesses at least five RNA structural elements including triplex-forming DNA-binding sites in structural motif I and two PRC2 binding sites for EZH2 and SUZ12 in structural motif III [91]. 3D modeling of the DNA–RNA triplex in motif I and PRC2 and lncRNA in motif III place them in close proximity, suggesting that they act in concert to execute H3K27me3 modification of chromatin. However, as best we can tell, imprinted genes within the *Gtl2* imprinted domain have not been assessed for triplex target sites.

The function of lncRNAs serving as guides leaves open many outstanding questions. Are there lncRNA specific sequences or 2D and/or 3D structural requirements for generating triplex formation and for modifier complex interactions? Four PRC2 proteins (EZH2, EED, SUZ12, and AEBP2) have known RNA-binding capacities [92–95] with high specificity to G-quadruplexes [96]. To anchor the RNA to chromatin and then target PRC2 to specific target regions, is RNA–DNA triplex formation required in conjunction with PRC2 binding to RNA? Do other chromatin modifying complexes also bind lncRNAs in a similar fashion? As multiple binding sites within a lncRNA align with different target sites within an imprinted domain, does this necessitate multiple copies of the imprinted lncRNA to target imprinted gene promoters or does the 3D structure of the lncRNA and of chromatin enable a single lncRNA to align with and silence multiple promoters simultaneously? In either case, does loss or interference with one target sequence simultaneously impact the alignment and silencing of other target sites?

### Imprinted lncRNAs as decoys

Another function attributed to lncRNAs is that of a molecular decoy. Decoy function is described by lncRNAs acting as a sink or sponge to sequester proteins away from a target site (Fig 2H). This could include transcription factors, signaling proteins, splicing factors, and chromatin modifying proteins or complexes [75,76]. At the *Gtl2* imprinted domain, the *Gtl2* lncRNA may function as a decoy (Fig 1F). In ES cells, blastocysts and neuronal cells, the maternal *Gtl2* ICR harbors an enhancer signature and produces bidirectional enhancer transcripts as well as binds AFF3 (AF4/FMR2 family member 3). AFF3 is a core component of the superelongation complex that facilitates an active chromatin state and promotes transcription of the polycistronic *Gtl2lt* transcript [24,97]. The *Gtl2* lncRNA, in turn, may act as a decoy for the *Gtl2* ICR, maintaining its active chromatin state [97,98]. This is supported by PRC2 and JARID2 interactions with the *Gtl2* lncRNA (which contrasts with weak binding by EZH2 and JARID2 and the lack of SUZ12 and H3K27me3 enrichment at the maternal *Gtl2* ICR) [88,99]. Upon maternal *Gtl2* promoter deletion, *Gtl2* lncRNA expression is abolished, leading to increased EZH2 binding at the *Gtl2* ICR [87]. Furthermore, following *Ezh2*, *Eed*, and *Jarid2* deletion, the maternal *Gtl2* ICR is occupied by DNMT3A and DNMT3L and becomes de novo methylated [97,98]. Together, the data suggest that the *Gtl2* lncRNA interacts with JARID2 and PRC2 to mitigate EZH2 methyltransferase activity and DNMT3A/3L recruitment at the maternal *Gtl2* ICR.

There are many outstanding questions about lncRNA decoy function. As a molecular decoy, lncRNAs may maintain the ICR in an active state and at the same time, the active ICR directs lncRNA expression. What controls this apparent feedback loop? How susceptible is the active ICR to fluctuations in lncRNA levels? Is decoy function mediated via RNA-binding proteins? What is the relationship between weak and strong chromatin modifier interactions with chromatin and lncRNAs? If lncRNAs act as decoys to titrate away positive and negative regulatory proteins, what mechanisms are in place to balance decoy versus other lncRNA function such as scaffold function?

### Imprinted lncRNAs as transregulators

In addition to imprinted lncRNAs operating in *cis* to regulate expression of imprinted genes within their respective domains, imprinted lncRNAs also regulate other genes or domains in *trans* [100]. Loss and gain of *H19* lncRNA function alter expression of imprinted genes in the *Kcnq1ot1*, *Airn*, *Nespas*, and *Gtl2* domains [101], while loss and overexpression of the human *IPW* lncRNA (from the *SNHG14* lncRNA) lead to changes in *GTL2* lncRNA, microRNA, and snoRNAs in the *GTL2* domain [102]. One factor that may contribute to lncRNAs *trans* function is that imprinted domains can colocalize to the same 3D nuclear space [103,104], generating imprinted gene networks [105], where expression of an imprinted gene in one domain influences expression of imprinted genes in other domains. While upstream or downstream regulatory pathways could indirectly account for these linked expression patterns [105], imprinted lncRNAs are likely to be directly associated with interactions between imprinted domains. With respect to nonimprinted genes, both *Gtl2* and *H19* lncRNAs are involved in the regulation of the signaling transforming growth factor β (TGF-β) pathway [106,107], the cell fate Wnt/β-catenin pathway [108,109], and the tumor suppressor p53 response pathway [110,111]. Overall, the *trans*-actions of these lncRNAs point to either lncRNA scaffold or guide function. As a scaffold function, the *H19* lncRNA interacts with the epigenetic modifier methyl-CpG-binding domain protein 1 (MBD1) and targets MBD1 to imprinted genes in *trans*, modulating repression through H3K9me3 [112]. In a similar fashion, *IPW* interacts with EHMT2, conferring H3K9me3 to the *Gtl2* ICR [102]. The *Gtl2* lncRNA interacts with JARID2 and PRC2, directing H3K27me3 to gene targets outside of the *Gtl2* domain [88]. As lncRNA guide function, bioinformatic predictions of *H19* lncRNA–DNA triplex binding sites in humans and mice identified promoter target sites at multiple imprinted genes, including *Igf2r*, *Gnas*, *Dlk1*, *Peg1*, and *Cdkn1c* [113], while the human *GTL2* lncRNA has DNA triplex binding sites that map to multiple genome-wide target sites in both human and mouse cell lines [107,114]. While the mechanisms of *H19* lncRNA regulation of gene targets in *trans* remain to be fully elucidated, the *Gtl2* lncRNA–DNA triplex structures may act together with its PRC2-binding motifs to modulate gene targets in *trans* [107].

As stated above, many outstanding questions regarding lncRNA scaffold and guide function remain to be resolved. More specifically for lncRNA *trans* function, what mechanisms operate for imprinted lncRNAs to find their targets? Is lncRNA *trans* function at imprinting gene networks and other potential chromosomal interactions left to random associations or are there active mechanisms to target imprinted lncRNAs to single gene promoters or to chromosomal networks?

## Emerging functions of posttranscriptional lncRNAs

### Imprinted lncRNAs as higher-order structures

lncRNAs are able to fold into secondary and tertiary higher-order 3D structures, confining them to the nucleus and influencing their regulatory function [115]. While the dynamics of RNA folding is an important component of human *GTL2* lncRNA function [111], the recently identified tertiary structure of the *GTL2* lncRNA has shed new light on the importance of higher-order tertiary structures [116]. Here, highly conserved motifs within the *GTL2* lncRNA interact to generate pseudoknot structures, with specific loops imparting a stable 3D structure (“kissing loops”) [116]. Surprisingly, point mutations to the conserved motifs disrupt formation of the pseudoknot and compromise *GTL2* lncRNA function in *trans*, which is independent of the ability of the *GTL2* lncRNA to recruit proteins, such as PRC2. To the best of our knowledge, imprinted genes within the *Gtl2* imprinted domain have not been assessed for the effects of altered *Gtl2* secondary and tertiary structure on their expression. Specific conserved motifs within *Kcnq1ot1* lncRNA also contribute to folding and secondary stem-loop structure of the lncRNA [80]. Mutations in this motif region perturbs the structure of *Kcnq1ot1* lncRNA and leads to a relaxation of paternal allelic silencing of imprinted genes within the domain [80]. Whether the *Kcnq1ot1* lncRNA forms tertiary structures to carry out its function remains to be determined.

The mechanism of how lncRNA higher-order structure contributes to its ability to regulate expression remains unknown. It has been postulated that spatial organization of lncRNA pilots their function by directing protein positioning and localizing lncRNAs to target sites [80,116]. Thus, is the tertiary-folded lncRNA involved in contributing to scaffold or guide function? If structural motifs with lncRNAs must be in close proximity to function, what would be the consequence of inserting a wedge between these structural motifs? Do all imprinted lncRNAs attain a higher-order, 3D structure?

### Imprinted lncRNAs as architectural components

Even with the above lncRNA functions, the question still remains as to how lncRNAs navigate upstream of the lncRNA gene to ultimately carry out long-range silencing. The answers to this question usually involve explanations of chromatin interaction generating active and repressed chromatin loops. Emerging evidence suggests that lncRNAs may play a role by facilitating chromatin interactions. The classic model of chromatin looping at imprinted domains is the *H19* imprinted domain. CCCTC-binding factor (CTCF) and the cohesin complex generate insulator activity and chromatin looping between the maternal *H19* ICR and downstream enhancer, which leads to upstream maternal *Igf2* silencing (Fig 1E) [117–119]. A recent analysis of topological-associated domains (TADs) at the *H19* and *Gtl2* domains revealed that while both parental domains possess the same CTCF-associated TAD organization, at the subTAD level, a maternal-specific CTCF site within the unmethylated *H19* ICR and *Gtl2* promoter bisected the TAD into two smaller subTADs [120]. This physically isolates the active maternal *H19* and *Gtl2* alleles from the silent maternal *Igf2*, and *Rtl1* and *Dio3* alleles, respectively. However, the maternal subTAD that encloses the *Gtl2* ICR and promoter, also includes the upstream, silent *Dlk1* allele. Thus, the simple scenario of active alleles in one chromosomal loop/subTAD and repressed alleles in another loop/subTAD is unsubstantiated at this domain, requiring additional mechanisms for allelic silencing for the upstream *Dlk1* allele. Since the *Gtl2* lncRNA prevents activation of the maternal *Dlk1* allele, it was proposed that the *Gtl2* lncRNA recruits CTCF to the maternal *Gtl2* promoter DMR though CTCF’s ability to bind RNA [121,122], focusing *Gtl2* lncRNA repressive function within the subTAD to the upstream *Dlk1* allele [120].

At the paternal *Kcnq1ot1* domain, the *Kcnq1ot1* lncRNA may function as an architectural component in chromatin looping. On the paternal allele, the *Kcnq1ot1* lncRNA interacts with and coordinates chromatin looping between the paternal *Kcnq1ot1* ICR and the *Kcnq1* promoter. Loss of *Kcnq1ot1* lncRNA through artificial methylation of the paternal *Kcnq1ot1* ICR, *Kcnq1ot1* promoter deletion, premature termination of the *Kcnq1ot1* lncRNA, or posttranscriptional depletion of *Kcnq1ot1* lncRNA disrupts chromatin interactions at the paternal *Kcnq1ot1* ICR and *Kcnq1* promoter in mouse embryonic fibroblasts (MEFs) and neonatal heart [123,124]. In MEFs, this results in paternal allelic reactivation of the downstream *Kcnq1* and upstream *Cdkn1c* genes [123]. These data point to a role for the *Kcnq1ot1* lncRNA in directing chromatin interactions that may generate an active loop for the *Kcnq1ot1* lncRNA gene while exclusion of paternal upstream and downstream alleles may lead to their repression. This role may be developmentally dependent, as posttranscriptional depletion of *Kcnq1ot1* lncRNA did not impact paternal allelic silencing of imprinted genes in ESCs and TSCs [50], and CTCF had little to no interaction with the *Kcnq1ot1* lncRNAs in TSCs [22].

At the *Airn* domain, chromatin-chromatin interactions are observed at the maternal *Airn* domain between the *Airn* gene body and the *Slc22a3* promoter in visceral yolk sac endoderm [41]. At the paternal domain, the *Airn* lncRNA disrupts these chromatin interactions, as demonstrated by premature truncation of the *Airn* lncRNA. Since cohesin complex and CTCF binding had little enrichment at the paternal domain, and CTCF had little to no interaction with the *Airn* lncRNA in TSCs [22], it is suggested that the paternal domain lacks paternal-specific chromatin loops, and that the *Airn* lncRNA lacks a role as an architectural component.

Many unresolved questions remain for lncRNAs as architectural components. Does CTCF bind (a subset) of imprinted lncRNAs? If so, do lncRNAs play a role in recruiting or biasing CTCF binding to lncRNA promoters and ICRs? Is there also a role for lncRNAs in inducing chromatin looping? What other lncRNA-protein or lncRNA-chromatin interactions could be involved in chromatin looping? Do imprinted lncRNA have chromatin-templating roles on which chromatin-chromatin interactions are mediated?

## Discussion

**Fig 3:**
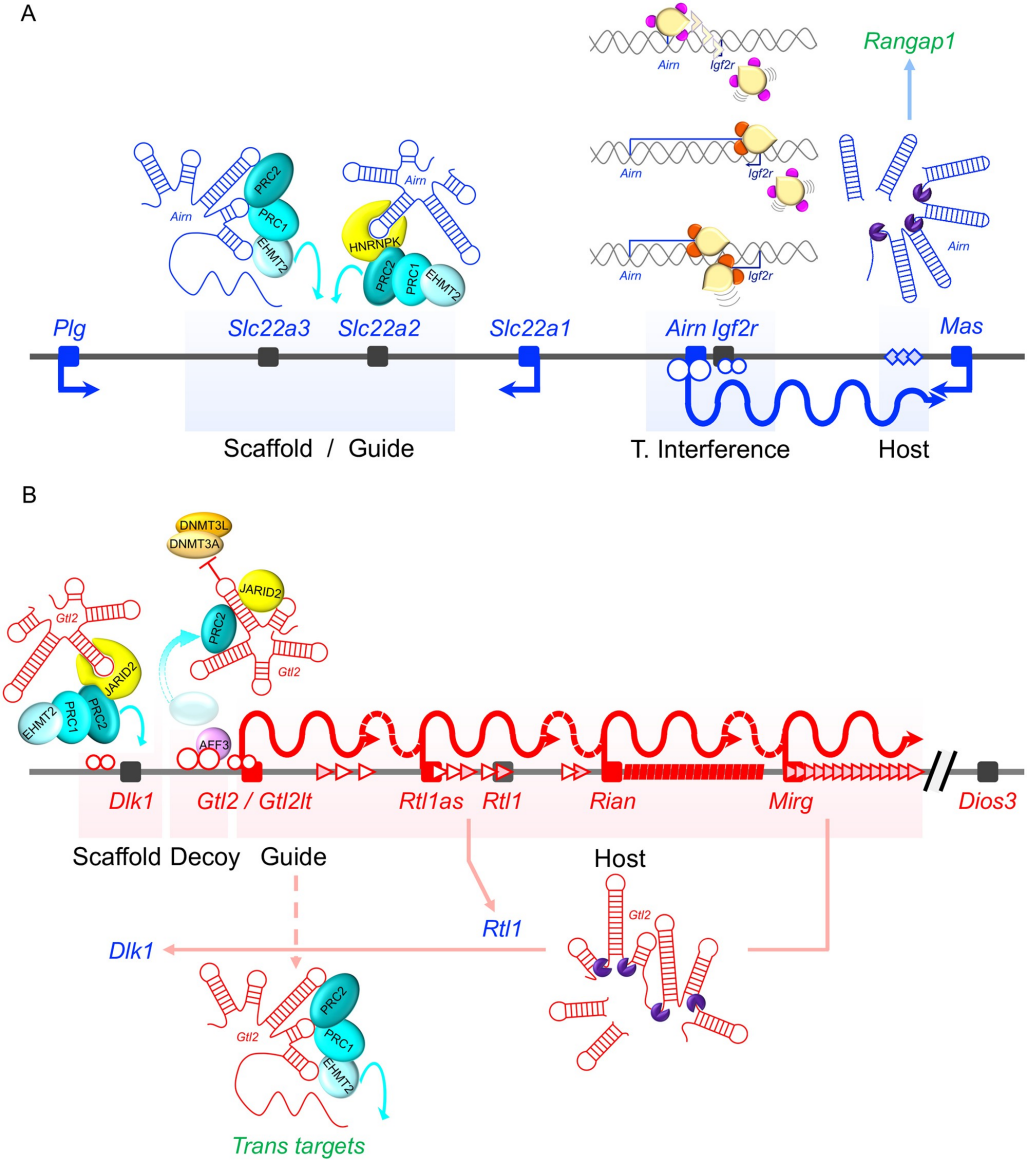
Multiplicity of function for lncRNA genes and their transcripts at (A) the paternal *Airn* imprinted domain and (B) the maternal *Gtl2* imprinted domain. Note that some lncRNA functions may be part of the same mechanism, for example, scaffold and guide function, incorporating higher-order lncRNA structure as well.

Since the discovery of the first imprinted lncRNA, significant progress has been made in understanding the functional roles of imprinted lncRNAs in imprinted domain regulation. Compelling evidence described above supports multiple functions for how lncRNAs regulate long-range repressive function. Perhaps the most remarkable feature is that these regulatory mechanisms are not mutually exclusive, with a single lncRNA and its transcription regulating different genes within an imprinted domain. For example, the paternal *Airn* lncRNA gene and its transcript act through transcriptional interference to silence the paternal I*gf2r* allele, as a host for small interfering RNAs, in long-range silencing of upstream paternal alleles through a lncRNA scaffold, and possibly as a guide to direct triplex formation (Fig 3A). By comparison, the maternal *Gtl2* lncRNA functions as a scaffold, a decoy, and a host for microRNAs and snoRNAs. It may also act through higher-order structure and as an architectural component upstream in *cis*, and as a guide in *trans* (Fig 3B). However, it is also possible that there is less multiplicity of lncRNA functions than the above findings indicate. For example, multiple mechanisms, such as lncRNA scaffold and guide function, may be different sides of the same coin, just investigated from different experimental paradigms. Alternatively, more recent, sophisticated, and detailed analyses may be a better reflection of lncRNA functionalities than earlier studies, requiring future experiments to retest past interpretations.

Moving forward, more in-depth, comprehensive studies are required to understand the properties of imprinted lncRNAs as well as to delve deeper into the actual mechanisms pertaining to imprinted lncRNA function and their mode of action. Case in point, to establish the plausibility of posttranscriptional lncRNA functions, it is essential to determine how imprinted lncRNAs are specifically localized to their *cis* and *trans* targets as well as the stoichiometric relationships between lncRNAs, protein partners, and their target sites [125,126]. With regard to emerging imprinted lncRNAs functions, it remains to be seen how broadly applicable they are across imprinted domains. At the very least, these emerging functions emphasize the complexity of imprinted lncRNA regulation and stress the need for continued research into potentially novel lncRNA functions. Furthermore, important questions remain regarding lncRNA regulation of imprinted domains in the context of developmental stage-specific and cell-type specific regulation. Saliently, imprinted lncRNAs and their long-range regulation of imprinted domains will continue to be a rich model for bettering our understanding of the mechanisms of lncRNA functionality and increasing our knowledge of lncRNA-associated pathophysiologies in human disease, which ultimately, may lead to medical advances in RNA therapeutics that target long noncoding RNAs.
